# Stroke patients treated by thrombectomy in real life differ from cohorts of the clinical trials: a prospective observational study

**DOI:** 10.1186/s12883-020-01653-z

**Published:** 2020-03-05

**Authors:** Milani Deb-Chatterji, Hans Pinnschmidt, Fabian Flottmann, Hannes Leischner, Anna Alegiani, Caspar Brekenfeld, Jens Fiehler, Christian Gerloff, Götz Thomalla

**Affiliations:** 1 Department of Neurology, University Medical Center Hamburg-Eppendorf, Martinistrasse 52, 20246 Hamburg, Germany; 2 Institute of Medical Biometry and Epidemiology, University Medical Center Hamburg-Eppendorf, Hamburg, Germany; 3 Department of Neuroradiological Diagnostics and Intervention, University Medical Center Hamburg-Eppendorf, Hamburg, Germany

**Keywords:** Stroke, Thrombectomy, Reperfusion, Basilar artery occlusion, Clinical practice

## Abstract

**Background:**

Randomized controlled trials (RCTs) demonstrated efficacy and safety of endovascular treatment (ET) in anterior circulation large vessel occlusions (LVO). We aimed at investigating how stroke patients treated by thrombectomy in clinical practice and their outcome compare to cohorts and results of thrombectomy trials.

**Methods:**

In a prospective study, we consecutively included stroke patients treated by thrombectomy (2015–2017). Baseline characteristics, procedural and outcome data were analyzed. Outcome was assessed by modified Rankin Scale (mRS) at 90 days. Ordinal regression analysis was performed to identify predictors of outcome.

**Results:**

Thrombectomy was applied in 264 patients (median 75 years, 49.6% female). Median baseline National Institutes of Health Stroke Scale (NIHSS) was 16, 58.0% received concomitant intravenous thrombolysis, 62.1% were referred from external hospitals. Median Alberta Stroke Program Early CT Score (ASPECTS) was 7. Successful recanalization (modified Thrombolysis in Cerebral Infarction Score, mTICI 2b/3) was achieved in 72.0%. Symptomatic intracranial hemorrhage (sICH) occurred in 4.5%. Independent outcome (mRS 0–2) was achieved in 26.2%, poor outcome (mRS 5–6) in 49.2%. Only 33.5% met the stringent enrolment criteria of previous RCTs. Lower age, baseline NIHSS, pre-stroke mRS, higher ASPECTS, and successful recanalization were independent predictors of favourable outcome.

**Conclusions:**

The majority of stroke patients treated by ET in clinical practice would not have qualified for randomization in prior RCTs. Outcome in real-life patient cohorts is worse than in the highly selected cohorts from randomized trials, while rates of successful recanalization, sICH and outcome predictors are the same. Our findings support ET in broader patient populations than in the RCTs and may improve treatment decision in individual stroke patients with LVO in clinical practice.

## Background

Several randomized controlled trials (RCT) demonstrated the beneficial effect of endovascular treatment (ET) in stroke patients with an anterior circulation large vessel occlusion (LVO) [1–5]. These results led to a paradigm shift in acute stroke treatment, mechanical thrombectomy now being standard of care for acute ischemic stroke due to LVO. However, in most of these trials strict inclusion criteria were applied restricting enrollment to a homogenous patient population with small stroke lesions, occlusion of the intracranial carotid artery or proximal middle cerebral artery (MCA, M1-segment), being treated within the first 6 h after symptom onset. Owing to the patient’s inclusion criteria and the results of these trials the American Stroke Association/American Heart Association (ASA/AHA) recommended ET in a selected patient group with acute ischemic stroke [6].

Recently, two RCTs, demonstrated efficacy of ET in patients with anterior circulation LVO beyond 6 h after symptom onset [7, 8], but again in both trials randomization was restricted to highly selected patients with imaging findings indicating high likelihood of treatment response, e.g. only small infarct core. Hence, there is still uncertainty about the benefit and risk of thrombectomy in patients not meeting the proposed ASA/AHA-criteria. There is also only limited data on how patients treated by thrombectomy in clinical practice comply with the ASA/AHA-criteria, and on outcome of thrombectomy in these patients.

Therefore, we studied procedural parameters and outcome of thrombectomy in a real-world prospective cohort of patients. We aimed to investigate how patients treated in clinical practice compare to patients from the RCTs. We further aimed to identify predictors of clinical outcome, and to compare outcome of the patients meeting ASA/AHA guideline criteria for thrombectomy to those not meeting these criteria.

## Methods

### Patients

All consecutive patients (18 years and older) with acute ischemic stroke who were treated by mechanical thrombectomy between July 2015 and June 2017 in our center were prospectively enrolled. Patients were either referred directly to our hospital (*mothership*), or transferred from external hospitals for ET (*ship*). The decision to treat was made interdisciplinary between a vascular neurologist and interventional neuroradiologist on a case-by-case basis guided by an institutional protocol. Factors taken into account for treatment decision included the elapsed time from symptom onset, stroke severity assessed by the NIHSS on admission, site of vessel occlusion, the ASPECT score, the pre-stroke disability, and extended imaging with CT-perfusion or MRI to identify potentially salvageable brain tissue, in particular, beyond 6 h of symptom onset or if the symptom onset time was uncertain. Patients received standard treatment including intravenous thrombolysis (IVT) prior to endovascular therapy if appropriate according to national and international guidelines.

### Clinical and radiological data

Baseline characteristics included demographical data, baseline NIHSS, the pre-stroke modified Rankin Scale (mRS) score, concomitant anticoagulation, pre-existing vascular risk factors and information on stroke onset time.

Additionally, the site of vessel occlusion – identified either by computed tomography (CT) or magnetic resonance imaging (MRI) - was recorded. The extent of early ischemic changes was assessed using the Alberta Stroke Program Early CT Score (ASPECTS) score on baseline imaging of all anterior circulation vessel occlusions. Recanalization result was defined using the modified Thrombolysis in Cerebral Infarction Score (mTICI). All patients received subsequent brain imaging within 24 h after intervention.

This study was conducted in accordance with ethical principles for human studies and approved by the local ethics committee. Written informed consent was obtained from the patient or the proxy. Consent was waived when patients died before consent could be obtained or lacked the capacity to give consent and no proxy was available.

### Procedural and outcome data

Mechanical thrombectomy was performed by six interventional neuroradiologists on a 24/7 basis. Different devices were used according to the preference of the interventional neuroradiologist.

Successful recanalization was defined as mTICI ≥2b. Symptomatic intracerebral hemorrhage (sICH) was defined as an occurrence of ICH combined with a deterioration of at least 4 points on NIHSS score or indication for surgery or death (according to the SITS-MOST criteria [9]). Time between symptom onset to groin puncture (SOG) and symptom onset to recanalization (SOR) were calculated.

Periprocedural and post-treatment complications were documented until discharge. A follow up was performed after 90 days assessing the mRS by telephone interview with the patients, the next of kin or caregiver. The assessors were blinded to patients’ variables including the results of ET.

### Statistical analysis

For between-group comparisons of categorical variables, χ^2^-tests or Fisher exact tests were used, as appropriate. Mann-Whitney-U tests were employed for continuous variables. Predictors of better outcome (lower mRS scores) were calculated by ordinal regression analyses on the mRS score at 90 days as the dependent variable. The variables sex, IVT, ship, successful recanalization (defined by mTICI 2b/3), anterior/posterior circulation, compliance with the ASA/AHA guidelines for thrombectomy in acute ischemic stroke patients (patients ≥18 years, pre-stroke mRS score of 0–1, internal carotid artery occlusion or MCA (M1), NIHSS of ≥6, ASPECTS of ≥6, treatment within 6 h after symptom onset, pre-treatment with IVT in eligible patients according to the guidelines from professional societies; ASA/AHA-group/non-ASA/AHA-group) and mRS before admission (categorized to 0–1 and 2–5) were considered as dichotomous independent variables. Side of vessel occlusion was considered as a multinomial variable (right, left, posterior circulation) and age, baseline NIHSS, SOR and the ASPECT score as continuous independent variables.

Univariate and multivariate ordinal regression analyses were applied. Multivariate analyses were conducted with a backward selection method. These analyses were run in three settings that differed with respect to the included variables contained in the initial model and to the total amount of patients in which complete information on the different variables was available:

1. An initial model containing the entire study sample with all independent variables (*n* = 243),
2. Subsequently, an initial model focusing on the anterior circulation infarcts with
  - the same independent variables plus the ASPECT score (*n* = 200),
  - the same independent variables plus the ASPECT score and SOR (*n* = 105). The variable ship was excluded from this subanalysis due to the high correlation between the variables ship and SOR.

The resulting odds ratios (OR) with 95% confidence intervals (CI) and *p* values are presented. *P* values < 0.05 were considered statistically significant. All tests were two-sided. The statistical analysis was performed using SPSS (Version 25.0; IBM, Armonk, New York).

## Results

### Patient characteristics

A total number of 264 patients received mechanical thrombectomy within the study period. Baseline characteristics are displayed in Table 1. One hundred sixty-four patients (62.1%) were referred for ET from external hospitals (*ship*), while 100 patients (37.9%) presented directly to our center (*mothership*). Median age was 75 years (IQR 64–81), 49.6% were female. Seventy patients (26.5%) aged older than 80 years. The median baseline NIHSS score was 16, 153 patients (58%) received IVT before ET. Vessel occlusions of the anterior circulation were present in 221 (83.7%) cases, whereas 43 (16.3%) subjects suffered from LVO in the posterior circulation. In patients with anterior circulation stroke, the median ASPECTS was 7. The distribution of the pre-stroke mRS scores of the entire study population is displayed on Additional file 1.

**Table 1:** Baseline characteristics (*n* = 264)

Age (years) - median (IQR)	75 (64–81)
Male – n (%)	133 (50.4)
Risk factors - n (%)	
Arterial hypertension	181 (68.6)
Hyperlipidemia	40 (15.2)
Diabetes mellitus	47 (17.8)
Atrial fibrillation	100 (37.9)
Stroke etiology – n (%)	
Atherosclerosis	109 (41.3)
ICA stenosis (> 70%)	40 (15.2)
Atrial fibrillation	119 (45.1)
Dissection	3 (1.1)
Other (e.g. endocarditis)	17 (6.4)
Undetermined	16 (6.1)
mRS before admission (*n* = 263) – median (IQR)	0 (0–1)
Baseline NIHSS – median (IQR)	16 (12–20)
Anticoagulation before admission – n (%)	54 (20.5)
Time of symptom onset unknown – n (%)	98 (37.1)
Wake up stroke	22 (8.3)
Unwitnessed stroke	63 (23.8)
Any time points not documented	13 (4.9)
Intravenous thrombolysis – n (%)	153 (58)
Ship – n (%)	164 (62.1)
Mothership – n (%)	100 (37.9)
*Imaging characteristics*	
ASPECT Score (*n* = 218) - median (IQR)	7 (6–9)
Site of vessel occlusion	
*Anterior circulation – n (%)*	
ICA-intracranial Carotid T	53 (20.1)
ICA- intracranial no Carotid T	10 (3.8)
ICA-extracranial	1 (0.4)
M1 proximal	79 (29.9)
M1 distal	43 (16.3)
M2	22 (8.3)
Tandem occlusion (ICA, MCA)	13 (4.9)
*Posterior circulation – n (%)*	
Basilar artery	39 (14.8)
Vertebral artery	3 (1.1)
PCA	1 (0.4)

*Abbreviations*: *IQR* Interquartile range, ACI, *mRS* Modified Rankin Scale, *NIHSS* National Institutes of Health Stroke Scale, *ASPECTS* Alberta Stroke Program Early CT Score, *ICA* Internal carotid artery, *M1* First segment of middle cerebral artery, *M2* Second segment of the middle cerebral artery, *PCA* Posterior cerebral artery

### Procedural and outcome parameters

In patients with known time of symptom onset, median time of SOG was 240 min (IQR 180–310), median time of SOR was 293 min (IQR 227–370) (Table 2). The time elapsed from SOG and SOR was longer in patients treated by *ship* (285 min, IQR 232–330 and 330 min, IQR 280–392, respectively) than by *mothership* (147 min, IQR 111–200 and 203 min, IQR 145–252, respectively).

**Table 2:** Procedural and outcome parameters

*Workflow times*	
Symptom onset to groin puncture (min) (*n* = 163) – median (IQR)	240 (180–310)
Symptom onset to recanalization (min) (*n* = 137) – median (IQR)	293 (227–370)
*Outcome parameters*	
mTICI 2b/3 – n (%)	190 (72.0)
Any ICH – n (%)	46 (17.4)
sICH – n (%)	12 (4.5)
mRS at 90 days (*n* = 248) – median (IQR)	4 (2–6)

*Abbreviations: mRS* Modified Rankin Scale, *NIHSS* National Institutes of Health Stroke Scale*, IQR* Interquartile range*, mTICI* Modified Thrombolysis in Cerebral Infarction Score, *ICH* Intracerebral hemorrhage*, sICH* Symptomatic intracerebral hemorrhage

Successful recanalization (TICI 2b/3) was achieved in 190 subjects (72%), 103 of them (39%) reached complete recanalization (defined by a TICI 3 score). In eleven cases (4.2%) of these the occluded artery was found to be already recanalized in angiography prior to any endovascular procedure. An overview of the devices used for thrombectomy is given in the Additional file 2.

Periprocedural complications occurred in 19 patients (7.2%) and comprised vasospasm, dissections, bleedings, distal emboli. Within 24 h after intervention and during the entire hospital stay 117 patients (44.3%) suffered from complications which encompassed recurrence of stroke, groin haematoma/pseudoaneurysm, myocardial infarction, ICH, malignant media infarction, hemicraniectomy, severe pneumonia and others (e.g. acute mesenteric ischemia with lactic acidosis, urinary tract infection leading to sepsis, cardiac decompensation, multi-organic failure etc). Any ICH on follow-up imaging was found in 46 patients (17.4%), sICH occurred in 12 cases (4.5%) [see Additional file 3].

At discharge, mortality rate was 16.7% (44 patients). In 17 patients (6.4%) death was attributable to the initial ischemic stroke syndrome (4.2% malignant brain infarction, 2.3% severe brainstem syndrome). sICH precipitated death in eight patients (3.0%). In 16 cases (6.1%) reasons of death were related to initial stroke (e.g. severe pneumonia, recurrent stroke, multi-organic failure, paraneoplastic bleeding disorder etc.), in three patients (1.1%) causes of death were not related to stroke (e.g. cardiac decompensation etc.). In 20 (45.5%) of the patients, who died during the hospital stay, recanalization was futile.

**Fig. 1:**
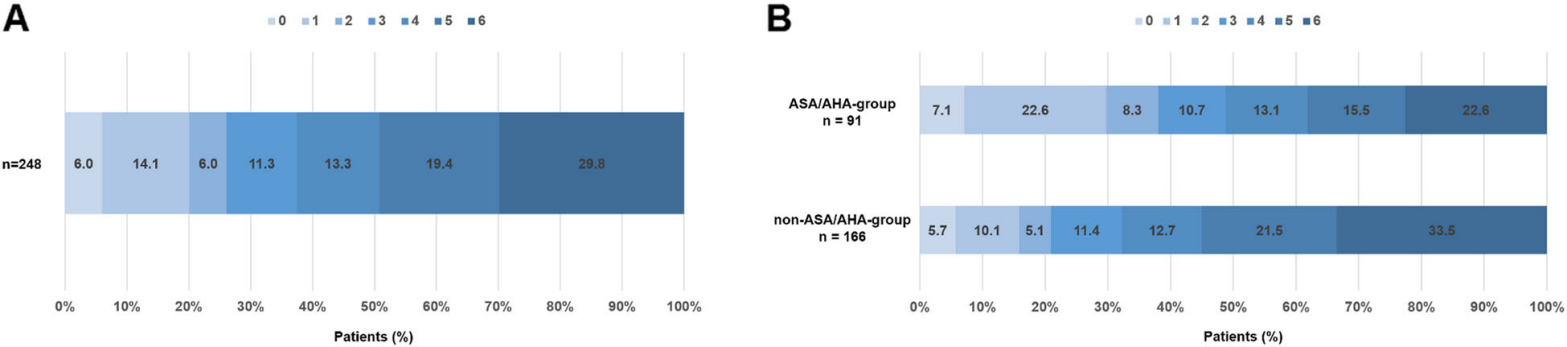
Distribution of the mRS scores 90 days after stroke. **a**: The distribution of the mRS scores 90 days after stroke in the entire patient cohort (*n* = 248) is displayed. Sixteen patients were lost to follow up. **b**: The distribution of the mRS scores 90 days after stroke in patients of the ASA/AHA-group (*n* = 91) compared to the non-ASA/AHA-group of patients (*n* = 166) is presented. Since detailed information was not available in seven patients of the study cohort, a classification of these patients in either group was not feasible

Follow-up information was available for 248 patients (94.0%). Independent outcome (mRS 0–2) was achieved in 65 cases (26.2%), while 122 patients (49.2%) suffered from a poor outcome (mRS 5–6). Mortality after 90 days was 29.8% (Fig. 1a).

### Predictors of clinical outcome

In multivariate analysis, lower age, lower pre-stroke mRS and lower baseline NIHSS scores, pretreatment with IVT, and successful recanalization remained as independent predictors of better clinical outcome (Table 3).

**Table 3:** Predictors of favourable outcome (multivariate ordinal regression analyses)

	OR^a^ (95% CI)	*P* values
*Entire study population (n = 243)*		
Age	0.950 (0.932–0.968)	< 0.001*
mRS before admission	0.333 (0.164–0.676)	0.002*
Baseline NIHSS	0.934 (0.910–0.958)	< 0.001*
Pretreatment with IVT	1.984 (1.261–3.121)	0.003*
TICI2b/3	4.202 (2.513–7.028)	< 0.001*
*Anterior circulation stroke including ASPECTS (n = 200)*		
Age	0.933 (0.912–0.955)	< 0.001*
mRS before admission	0.351 (0.161–0.764)	0.008*
Baseline NIHSS	0.877 (0.835–0.922)	< 0.001*
ASPECTS score	1.226 (1.052–1.428)	0.009*
TICI2b/3	4.893 (2.774–8.628)	< 0.001*
*Anterior circulation stroke including ASPECTS and symptom onset-to-recanalization (n = 105)*		
Age	0.937 (0.908–0.968)	< 0.001*
Baseline NIHSS	0.889 (0.822–0.962)	0.003*
ASPECTS score	1.360 (1.108–1.670)	0.009*
TICI2b/3	3.285 (1.208–8.935)	0.003*

*Abbreviations: OR* Odds ratio*, CI* Confidence interval*, mRS* Modified Rankin Scale*, NIHSS* National Institutes of Health Stroke Scale*, IVT* Intravenous thrombolysis, *mTICI* Modified Thrombolysis in Cerebral Infarction Score*, ASPECTS* Alberta Stroke Program Early CT Score

* significant

^a^OR > 1 indicate lower mRS scores at 90 days

In anterior circulation infarcts, after adding the variable ASPECTS to the analysis, lower age, lower pre-stroke mRS score, lower baseline NIHSS score, successful recanalization, and the ASPECT score were predictors of favourable outcome.

After including the variable SOR to this analysis, multivariate analysis revealed a lower age, lower baseline NIHSS scores, a higher ASPECT score, and a successful recanalization as predictors of good clinical outcome, while SOR was not.

An additional multivariable regression analysis including the interaction term (TICI2b/3 x ASA/AHA-group or non-ASA/AHA-group) showed no significant effect of the interaction term in the three models: entire study population (*p* = 0.89), patients with available ASPECTS (*p* = 0.88), patients with available ASPECTS and information on time to recanalization (*p* = 0.95).

### Application of the proposed ASA/AHA-criteria – a comparative analysis

A total number of 257 patients were able to be classified either to the group of patients complying with the suggested guidelines of the ASA/AHA or to those who did not adhere to these criteria (Table 4). In seven patients an allocation was not possible owing to the lack of information on time of symptom onset (*n* = 6) or ASPECTS (*n* = 1).

**Table 4:** Baseline characteristics, interventional and outcome parameters according to the proposed criteria of the ASA/AHA (*n* = 257)

	ASA/AHA-group *n* = 91	Non-ASA/AHA-group *n* = 166	*P* values
*Baseline characteristics*			
Age (years) - median (IQR)	74 (63–80)	75 (64–81.25)	0.439
Male – n (%)	46 (50.5)	86 (51.8)	0.847
Risk factors - n (%)			
Arterial hypertension	58 (63.7)	117 (70.5)	0.267
Hyperlipidemia	17 (18.7)	23 (13.9)	0.307
Diabetes mellitus	16 (17.6)	29 (17.5)	0.982
Atrial fibrillation	41 (45.1)	53 (31.9)	0.037*
Stroke etiology – n (%)			0.356
Atrial fibrillation	45 (49.5)	69 (41.6)	
Atherosclerosis	35 (38.5)	72 (43.4)	
ICA stenosis (> 70%)	20 (22.0)	20 (12.0)	
Dissection	2 (2.2)	1 (0.6)	
Other	6 (6.6)	11 (6.6)	
Undetermined	3 (3.3)	13 (7.8)	
Anticoagulation before admission – n (%)	25 (27.5)	25 (15.1)	0.016*
Baseline NIHSS – median (IQR)	16 (13–19)	16 (11–21)	0.864
mRS before admission - median (IQR)	0 (0–0)	0 (0–1) (*n* = 165)	< 0.001*
Ship – n (%)	62 (68.1)	101 (60.8)	0.246
Intravenous thrombolysis – n (%)	58 (63.7)	95 (57.2)	0.309
*Imaging characteristics*			
ASPECTS – median (IQR)	8 (7–9)	7 (5–8)(*n* = 121)	< 0.001*
*Workflow times*			
Symptom onset to groin puncture (min) – median (IQR) (*n* = 74)	235 (187–291) (*n* = 78)	250 (176–334) (*n* = 84)	0.118
Symptom onset to recanalization (min) – median (IQR) (*n* = 64)	284 (215–342) (*n* = 65)	318 (248–400) (*n* = 71)	0.024*
*Procedural and outcome parameters*			
mTICI 2b/3 – n (%)	68 (74.7)	117 (70.5)	0.469
ICH – n (%)	15 (16.5)	30 (18.1)	0.749
sICH – n (%)	3 (3.3)	8 (4.8)	0.751
mRS at 90 days – median (IQR)	4 (1–5) (*n* = 84)	5 (3–6) (*n* = 158)	0.006*

*Abbreviations: ASA* American Stroke Association*, AHA* American Heart Association*, NIHSS* National Institutes of Health Stroke Scale*, IQR* Interquartile range*, ASPECTS* Alberta Stroke Program Early CT Score*, mRS* Modified Rankin Scale*, mTICI* Modified Thrombolysis in Cerebral Infarction Score*, ICH* Intracerebral hemorrhage*, sICH* Symptomatic intracerebral hemorrhage

* significant

When applying the ASA/AHA guideline criteria for patient selection, only 91 patients (35.4%) met these criteria, while 166 patients (64.6%) did not comply with at least one of these criteria for thrombectomy: in 65 patients (39.1%) vessel occlusion occurred in the distal segment of the MCA (M2) or in the posterior circulation, 10 patients (6.0%) had a baseline NIHSS score < 6, 35 patients (21.1%) had an ASPECTS < 6, 37 patients (22.3%) suffered from pre-stroke disability with mRS > 1, 57 patients (34.3%) were treated beyond the 6 h threshold, and in 21 cases (12.7%) stroke occurred during sleep (wake up stroke).

As compared to patients complying with the ASA/AHA-guidelines, those not meeting these criteria showed a higher pre-stroke mRS score (*p* < 0.001), had a lower median ASPECTS (7 vs 8; *p* < 0.001), showed a longer time of SOR (318 min vs 284 min; *p* = 0.024), had a lower rate of good clinical outcome defined by mRS 0–2 (20.9% vs. 38.1%; *p* = 0.004), a higher rate of poor outcome assessed by mRS 5–6 (55.1% vs. 38.1%; *p* = 0.012) and a higher median mRS score at 90 days (5 vs 4; *p* = 0.006) (Fig. 1b). The mortality rate did not differ between these two subgroups.

## Discussion

In this prospective observational study, we studied clinical characteristics and outcome of stroke patients treated by thrombectomy in clinical practice. At first sight, our study population showed a higher mortality rate (29.8%), a higher rate of patients with poor outcome (49.2%), and a lower rate of patients achieving a good outcome (26.2%), as compared to the “big five” RCTs of thrombectomy in acute ischemic stroke (mRS 6: 9–18.4%, mRS 5–6: 17–30.1%, mRS 0–2: 32.6–71.7%, respectively) [1–5, 10] and the HERMES meta-analysis of these RCTs (mRS 6: 15.3%, mRS 5–6: 21.5%, mRS 0–2: 46%, respectively) [10]. However, there are several aspects in which our patient cohort differs from that of the RCTs, which applied strict inclusion criteria to randomize patients.

First, with a median age of 75 years patients in our population were older than in the HERMES meta-analysis (median age 68 years) [10]. One out of four patients in our sample was above the age of 80 years. It is well known, that following thrombectomy mortality rates are higher [11] and outcome is worse in elderly patients [12]. In line with this, our analysis identified a younger age as a consistent predictor of better outcome at 90 days, with an odds ratio of 0.950 reflecting a 5% decreased likelihood of a good outcome for every year of increase in age.

Second, patients treated by thrombectomy in our study had larger baseline ischemic stroke cores indicated by a median ASPECTS of 7 being lower than the median ASPECTS of 9 in the pooled analysis of the HERMES collaboration [10]. Higher pre-treatment ASPECT score is a known predictor of good outcome after stroke thrombectomy [13], a finding that was reproduced in our analysis. An increase of ASPECTS by one score point was associated with an odds ratio of 1.226 for a lower score on the mRS at 90 days. A considerable proportion of our study population (21.1%) showed large pretreatment infarct cores (ASPECTS < 6), which would have excluded patients from the majority of the RCTs [14].

Moreover, 14% of our study sample had pre-existing disability reflected by a pre-treatment mRS score > 1. This, again, would have excluded patients from most thrombectomy trials. And again a higher mRS score representing pre-stroke disability was identified as an independent predictor of worse outcome in our analysis.

Sixteen percent of the patients presented with a vessel occlusion located in the posterior circulation. LVO of the posterior circulation were excluded from the thrombectomy trials, thus it remains elusive to what extent this important subgroup of patients benefits from ET. While several studies already indicate that thrombectomy may be a safe and effective treatment modality in basilar artery occlusion (BAO) [15, 16], a significant higher mortality rate was reported in BAO as compared to anterior circulation stroke [17].

It becomes evident, that a large proportion of patients treated by thrombectomy under routine conditions in a large neurovascular center does not comply with the narrow inclusion criteria of the randomized trials. Indeed, almost two third of our patients would not have met the suggested ASA/AHA-criteria for thrombectomy. A similarly high proportion of non-ASA/AHA-patients was included in the TREVO Stent-Retriever Acute Stroke (TRACK) registry further supporting the hypothesis that in real life only a small group of patients matches the ASA/AHA guidelines [18]. Similar results were provided in another recently published single center analysis [19].

When comparing the patient groups meeting the ASA/AHA-criteria with those failing to match these criteria, we observed better outcome in the ASA/AHA-group of patients with 38.1% showing an independent outcome (mRS 0–2) after 90 days as compared to only 20.9% in the non-ASA/AHA-group. This is very likely explained by the fact that the suggested ASA/AHA-criteria, in the same way as the inclusion criteria of the RCTs, select those patients with the highest likelihood of treatment benefit. This approach was previously referred to as “cherry picking” of patients of the large thrombectomy trials [20]. While trials in highly selected populations are well suited to provide a clear demonstration of treatment benefit as a proof-of-concept, in clinical practice these results must be transferred to treatment decisions in individual patients with characteristics different from the trial cohorts.

Of note, in our entire patient cohort the recanalization rate (72.0%) as well as the rate of sICH (4.5%), as one of the most important safety outcome parameter, were within the range of the RCTs (58.7–88.0% and 3.3–7.7%, respectively) [1–5, 10] and comparable to the values of the HERMES meta-analysis (71 and 4.4%, respectively) [10]. Moreover, the delay from stroke onset to arterial puncture (240 min) or flow restoration (293 min) were similar to that of the RCTs [10]. These findings indicate, that in real life technical success and process parameters of thrombectomy comparable to those in the randomized trials can be achieved. In addition, the rate of successful recanalization, sICH, and mortality were comparable between the ASA/AHA- and the non-ASA/AHA-group of patients. This further supports the hypothesis that technical success and safety of ET in patients not meeting the strict trial criteria may be similar to a highly selected population, while the lower rate of good outcome relates to the poor predictive parameters, such as larger infarct cores, later treatment, or pre-stroke disability, inherent to this group.

Notably, we did not observe an interaction between successful recanalization and compliance with the ASA/AHA-group. Although interaction analyses might be underpowered, this finding suggests that the effect of achieving successful recanalization with regard to functional outcome is homogenous across both subgroups of patients (ASA/AHA eligible vs. non-eligible). Furthermore, regression analysis identified the same predictors of better outcome in both subgroups of patients, i.e. lower age, lower pre-stroke mRS, lower baseline NIHSS score, and successful recanalization (data not shown). In addition, compliance with the ASA/AHA-group of patients did not predict an independent outcome 90 days after stroke in our study population.

We also performed a systematic analysis of death after thrombectomy and of severe complications that occurred during in-hospital stay. Beside cerebral causes of death, i.e. malignant brain infarction, severe non-cerebral complications, such as pneumonia, mesenteric infarctions with lactic acidosis, cardiac decompensation, or multi-organic failure, were causes of death. A reason for the high rate of severe complications might be rooted in the large proportion of elderly patients with pre-existing comorbidities in our study population.

For clinical practice, these findings indicate, that although overall outcome may be worse in stroke patients not meeting the strict criteria of randomized trials or ASA/AHA guidelines, thrombectomy may as well be beneficial in these patients and less aggressive treatment may lead to a worse outcome. This is supported by results of the individual patient data meta-analysis by the HERMES collaboration that showed a benefit of ET in some of these subgroups [10], e.g. patients > 80 years, patients treated more than 6 h after symptom onset etc., while statistical power was limited owing to the low patient number. The facts, that in our cohort successful recanalization was a predictor of better outcome in the non-ASA/AHA group and the effect of achieving successful recanalization did not differ in both subgroups of patients in the interaction analyses, provide additional proof of the potential benefit of thrombectomy in non-ASA/AHA-eligible patients. For specific subgroups under-represented in the previous trials, further RCTs will be required to confirm the benefit of thrombectomy.

This study is limited by patients derived from a single center and lacking of a group of stroke patients with LVO not treated by ET. However, this study comprises a high number of prospectively enrolled stroke patients in clinical practice that were treated by ET in a center with a big catchment area. Hence, together with our findings available evidence strongly suggests that thrombectomy is feasible and safe in patients beyond those meeting the strict criteria of previous RCTs and ASA/AHA-recommendations, but the effectiveness needs to be confirmed in future RCTs.

## Conclusions

In a real-world setting the majority of stroke patients undergoing thrombectomy is treated outside the core inclusion criteria of the RCTs of stroke thrombectomy. Outcome of unselected patients treated in the “real world” is worse than in the highly selected patient cohorts of clinical trials owing to clinical characteristics that are associated with worse outcome (e.g., older age, larger infarct cores). However, rates of successful recanalization, sICH, and parameters predicting better outcome after ET are the same in these patients. By this, our findings support the application of thrombectomy in broader patient populations not represented in the RCTs.

## Supplementary information

**Additional file 1.** Distribution of the pre-stroke mRS score (*n* = 263). The distribution of the mRS scores before admission of the patients enrolled in this study is displayed on this table.

**Additional file 2.** Different applied devices. An overview of the devices applied during intervention is given in this table.

**Additional file 3. **Complications – periprocedural and during the hospital stay (*n* = 264). This table provides data on the complications which occurred during intervention and during hospital stay.

## Data Availability

All data generated or analysed during this study are included in this published article [and its supplementary information files].

## Abbreviations

ASA/AHA: American Stroke Association/American Heart Association
ASPECTS: Alberta Stroke Program Early CT Score
CT: Computed tomography
ET: Endovascular treatment
IQR: Interquartile range
IVT: Intravenous thrombolysis
LVO: Large vessel occlusion
MCA: Middle cerebral artery
MRI: Magnetic resonance imaging
mRS: Modified Rankin Scale
mTICI: Modified Thrombolysis in Cerebral Infarction Score
NIHSS: National Institutes of Health Stroke Scale
RCT: Randomized controlled trial
sICH: Symptomatic Intracerebral hemorrhage
SOG: Symptom onset to groin puncture
SOR: Symptom onset to recanalization
TRACK: TREVO Stent-Retriever Acute Stroke registry
