# Supplementary material for: Stroke patients treated by thrombectomy in real life differ from cohorts of the clinical trials: a prospective observational study
Source: BMC Neurol. 2020 Mar 5;20:81. doi: 10.1186/s12883-020-01653-z (PMC7059360; doi:10.1186/s12883-020-01653-z)
Supplement: Supplementary file 1 — Additional file 1. Distribution of the pre-stroke mRS score (n = 263). The distribution of the mRS scores before admission of the patients enrolled in this study is displayed on this table. [file 12883_2020_1653_MOESM1_ESM.docx]

**Additional file 1: Distribution of the pre-stroke mRS score (n=263)**

| *Pre-stroke mRS score – n (%)* | |
| --- | --- |
| 0 | 174 (66.2) |
| 1 | 53 (20.2) |
| 2 | 22 (8.4) |
| 3 | 5 (1.9) |
| 4 | 7 (2.7) |
| 5 | 2 (0.8) |

*Abbreviation: mRS, modified Rankin Scale*In one patient the mRS score before admission was not determinable.
