# Supplementary material for: Stroke patients treated by thrombectomy in real life differ from cohorts of the clinical trials: a prospective observational study
Source: BMC Neurol. 2020 Mar 5;20:81. doi: 10.1186/s12883-020-01653-z (PMC7059360; doi:10.1186/s12883-020-01653-z)
Supplement: Supplementary file 2 — Additional file 2. Different applied devices. An overview of the devices applied during intervention is given in this table. [file 12883_2020_1653_MOESM2_ESM.docx]

**Additional file 2: Different applied devices**

| *Devices* | |
| --- | --- |
| Stent-retrievers alone – n (%) | 124 (47) |
| Aspiration catheters alone – n (%) | 43 (16.3) |
| Combination of aspiration catheters and stent-retrievers – n (%) | 70 (26.5) |
| No devices applied (already recanalized vessel in angiography etc.) – n (%) | 27 (10.2) |
