# Supplementary material for: Stroke patients treated by thrombectomy in real life differ from cohorts of the clinical trials: a prospective observational study
Source: BMC Neurol. 2020 Mar 5;20:81. doi: 10.1186/s12883-020-01653-z (PMC7059360; doi:10.1186/s12883-020-01653-z)
Supplement: Supplementary file 3 — Additional file 3. Complications – periprocedural and during the hospital stay (n = 264). This table provides data on the complications which occurred during intervention and during hospital stay. [file 12883_2020_1653_MOESM3_ESM.docx]

**Additional file 3: Complications – periprocedural and during the hospital stay (n=264)**

| *Periprocedural complications (n=19)* | |
| --- | --- |
| Vasospasm – n (%) | 5 (1.9) |
| Dissections – n (%) | 6 (2.3) |
| Bleeding – n (%) | 8 (3.0) |
| Distal embolization – n (%) | 2 (0.8) |
| *Complications during hospital stay (n=117)* | |
| Recurrence of stroke – n (%) | 9 (3.4) |
| Groin haematoma/pseudoaneurysm – n (%) | 3 (1.1) |
| Myocardial infarction – n (%) | 3 (1.1) |
| Malignant MCA infarction – n (%) | 23 (8.7) |
| Hemicraniectomy – n (%) | 9 (3.4) |
| Pneumonia | 37 (14.0) |
| Any ICH – n (%) | 46 (17.4) |
| sICH | 12 (4.5) |
| Others | 76 (28.8) |

*Abbreviations: MCA, middle cerebral artery; ICH, Intracerebral hemorrhage; sICH, symptomatic ICH.*
